# A Miniaturized Device Coupled with Digital Image Correlation for Mechanical Testing

**DOI:** 10.3390/mi13112027

**Published:** 2022-11-19

**Authors:** Daniel J. Cruz, Jose Xavier, Rui L. Amaral, Abel D. Santos

**Affiliations:** 1 INEGI, Institute of Science and Innovation in Mechanical and Industrial Engineering, Campus da FEUP, R. Dr. Roberto Frias 400, 4200-465 Porto, Portugal; 2 UNIDEMI, Department of Mechanical and Industrial Engineering, NOVA School of Science and Technology, Universidade NOVA de Lisboa, 2829-516 Caparica, Portugal; 3 LASI, Intelligent Systems Associate Laboratory, 4800-058 Guimarães, Portugal; 4 FEUP, Faculty of Engineering, University of Porto, R. Dr. Roberto Frias, 4200-465 Porto, Portugal

**Keywords:** miniaturized specimen, digital image correlation (DIC), Small Specimen Test Techniques (SSTT), Bauschinger effect, sheet metal characterization

## Abstract

Miniaturized mechanical testing based on small sample testing technology is a powerful technique to characterize the mechanical properties of different materials, and it is being used in different application fields. However, the small size of the specimens poses several challenges because the results are highly sensitive to measurement accuracy and the corresponding mechanical properties can change substantially due to the so-called specimen size effect. In this work, a novel testing device based on miniaturized specimens is presented. The equipment is designed to test materials in tensile and compressive loadings, but it is also capable of performing reverse-loading tests. Buckling of the specimen is an inherent phenomenon in compression loadings, especially for thin materials. Therefore, specimen geometry is properly studied and optimized to mitigate this effect. To evaluate the deformation of the specimen, the digital image correlation (DIC) technique is used to capture the full-field strain in the central gauge section of the sample. A sensitivity analysis of the DIC setting parameters was performed for this application. To evaluate the performance of the developed system, experimental results of monotonic tests and tests with reverse loadings (tension-compression) are presented, considering two high-strength steels (DP500 and DP780).

## 1. Introduction

The mechanical behavior of engineering materials is typically determined by experimental tests using the so-called “macro specimens”, with dimensions on the scale of millimeters. However, in some applications, the material to be characterized is not available in the form of large blocks [1], which has recently motivated an increasing interest in evaluating the mechanical properties of materials with “miniaturized” specimens, also known by other names such as “small-size”, “sub-size”, or “mini” specimens [2,3]. Miniaturized specimens were first developed in the 1970s to characterize materials for in-service nuclear reactor pressure vessels [4]; this is an application in which surveillance capsules are used to periodically monitor the degradation of vessel materials. These capsules typically have limited storage space, and for this reason, a limited specimen size is required [5]. As a result, Small Specimen Testing Technology (SSTT) has been developed and applied in different application fields such as residual life assessment of in-service components [6], local measurement of properties in the welding industry [7,8], the additive manufacturing process [9,10], and the evaluation of mechanical properties of miniature devices such as microelectromechanical systems (MEMS) [11,12].

Several non-standard test techniques are currently available to evaluate the mechanical behavior of materials using miniaturized specimens [3]. One of the most used SSTT is the miniaturized tensile test (MTT) [13], which uses specimens with dog-bone geometry with nominal dimensions ranging from several millimeters to hundreds of microns. No standards have yet been established for MTT, which has resulted in different specimen designs. The design of miniaturized specimens currently uses one of two approaches: (1) simply scaling down from standard specimens, or (2) scaling down from standard specimens, with a subsequent adjustment of some dimensions. A comprehensive review of this topic can be found in the recent work of [2].

Miniaturized tensile testing encompasses several challenges related to specimen manufacturing, handling, and procedural issues. In addition, the miniaturization may cause the so-called “specimen size effect”, which leads to different behaviors at the miniaturized and macro scales. Specimen size effects can be categorized into two different levels. At the first level, the test results obtained from miniaturized specimens reflect the bulk mechanical properties described by continuum mechanics. However, for some materials, the results may deviate from standard specimens, and additional data processing techniques, e.g., the inverse Finite Element Method (FEM), are required [14,15,16]. On the other hand, at the second level, the specimen can no longer reflects the bulk properties as the thickness or diameter is further decreased. Typically, the critical thickness value is defined by the ratio t/d between thickness, *t*, and grain size, *d*, of the material. Generally, miniaturized specimens with a t/d ratio between 6 and 10 can achieve standard bulk behavior.

Sheet metal forming is an important manufacturing process in the production of metal components, applied in a wide variety of industrial areas such as automotive, naval, electrical, aeronautics, and aerospace [17]. Sheet metal forming includes simple processes such as bending, stretch forming and spinning, as well as more complex ones such as roll forming and deep drawing. In these processes, material behavior assessment is crucial for the correct calibration of material constitutive models. Therefore, a wide variety of tests are established in the literature to obtain reliable experimental data used in the identification of constitutive parameters.

The mechanical characterization of metallic sheets has standardized test procedures, by using the tensile test (uniaxial loading), according to ISO 6892-1 [18] and the hydraulic bulge test (biaxial loading), according to ISO 16808 [19]. On the other hand, no standardized test setup has yet been established to characterize the compressive loading of sheet metal materials [20]. Moreover, an uniaxial compressive stress state may cause buckling in flat sheet metal materials for small compressive strains. Therefore, the test method becomes invalid due to the change on the stress state. Over the past few years, different approaches have been developed to minimize buckling during compression tests. These approaches can be categorized into three groups: (i) the pack method, which involves packing a group of samples together in the thickness direction; (ii) a single sample with a lateral support system; and (iii) miniaturized test samples with a low gauge length/thickness ratio.

The pack method, developed by Aitchison et al. [21] and later improved by Jackman [22], involves joining together a group of samples along the thickness direction to form a unique specimen. With this strategy, the length/thickness ratio is reduced, and therefore the buckling instability under compression is minimized. A variation in the pack method was used by Yoshida et al. [23], in which five specimens were glued together and then covered with clamping plates. Because the pack method requires a large number of specimens and complex preparation, some authors prefer to use a single sheet with a lateral support system. Boger et al. [24] sandwiched a single flat specimen between two solid support plates using a constant restraining side force. To prevent buckling in the thickness direction (T-buckling), width direction (W-buckling), and unsupported gap (L-buckling), an oversized dog-bone specimen was designed. A device with two sets of fork-shaped supports was proposed in the same year by Sekine and Kuwabara [25] to eliminate the uncovered area in tensile-compressive relative movements. Because the male and female dies slide past one another as the sample is compressed, this design made it possible to support the entire length of the specimen.

The gauge-length/thickness ratio of the specimen has the biggest impact on the buckling phenomenon in compression. In order to effectively study the compression behavior of different sheet metal materials, test methods using miniaturized specimens have recently been suggested. Tritschler et al. [26], tested a titanium alloy with a thickness of 1.2 mm in compression using specimens with dimensions of 2.5 mm in length by 2 mm in width, achieving about 2% compressive strains. Hußnätter [27] used a specimen geometry of 2 mm length by 2 mm width to characterize the compressive behavior of magnesium alloy AZ31, achieving about 5% compressive strains before the specimen buckles.

Cruz et al. [28] developed an experimental equipment to perform uniaxial mechanical tests on miniaturized metallic specimens. The current work is a step forward in these developments, exploring the applicability of this equipment to describe the mechanical behavior of sheet metal materials, not only for monotonic uniaxial tests, i.e., tension and compression, but also for reverse loading tests, i.e., tension followed by compression or vice versa. Moreover, the digital image correlation technique was coupled with the miniaturized test device in order to quantify the quality of the test method regarding the specimen deformation at the scale of observation, in a balance between spatial resolution and accuracy [29].

## 2. Materials and Methods

### 2.1. Materials

**Figure 1:**
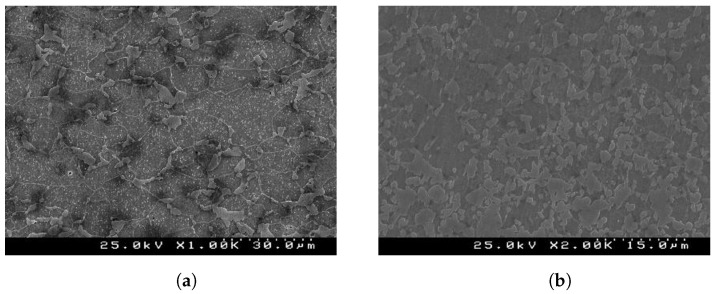
Initial microstructure obtained from SEM of (**a**) DP500 and (**b**) DP780 dual-phase steel.

In the present work, advanced high-strength steels (AHSS) produced by SSAB company were used, namely two grades of dual-phase (DP) steel sheets (DP500 and DP780) with an initial thickness of 0.8 mm. The dual-phase (DP) steel is an advanced high-strength steel (AHSS), with a good balance of strength and ductility due to its martensite islands in a ferrite matrix, as represented in Figure 1. Table 1 lists the percentage of alloying present in each material chemical composition.

### 2.2. Design of an Uniaxial Test Equipment for Miniaturized Specimens

The experimental test procedure for miniaturized specimens followed a similar procedure to the one typically carried out for conventional standard specimens, which is based on moving grips and results of force as a function of elongation/strain data. However, a very accurate procedure is required because results can be significantly affected by the testing protocol, including sample preparation, test setup, experimental operation, measurements, and data processing [2]. Several authors have emphasized different aspects to be taken into account when testing miniaturized specimens [3]: (a) the importance of the specimen-fabrication technique, which can have consequences for the measured properties, particularly on the yield strength; (b) good control and accuracy of the specimen dimensions, which directly relate to the reliability and reproducibility of the test data; (c) high-quality measuring instruments; and (d) the importance of a precise and well-aligned test frame, in order to minimize the extraneous bending strains.

**Figure 2:**
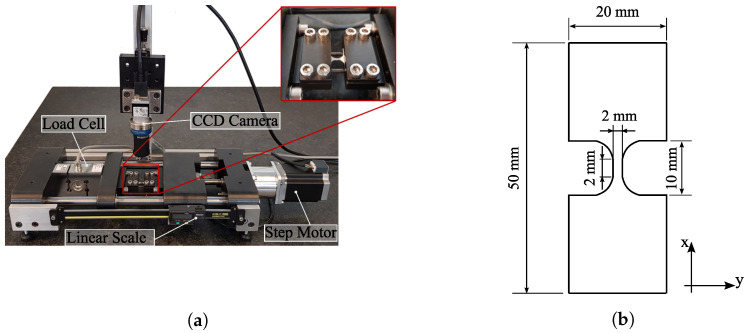
Miniaturized Specimen Tester Device (MSTD): (**a**) setup, and; (**b**) geometry of the miniaturized specimen.

Based on these considerations, a new type of equipment was developed, specifically designed to test miniaturized specimens on a reduced scale, and we refer to it as a Miniaturized Specimen Tester Device (MSTD) [28]. As represented in Figure 2, MSTD consists of three groups of components with their well-defined function. Firstly, there is the “motor group”, which includes a ball screw, driven by a stepper motor, which guarantees continuous and precise movement of the specimen, making it possible to guarantee a minimum displacement of 2 µm for each step of the motor. Secondly, there is the “frame group”, which ensures the layout and guidance of the various components, consisting of four linear bearings that slide on two ground shafts. Finally, there is the instrumentation group, consisting of a 5 kN “S”-type load cell and two extension measurement components: a linear ruler for measuring and controlling the positioning of the moving table and a digital image correlation (DIC) system. The MSTD’s layout allows all components to be on a common plane, which is advantageous for a precise alignment of the specimen during the test. Consequently, the appearance of undesirable bending moments is minimized, especially in compression, which is also essential to prevent the buckling of the specimen. The equipment, designed to test miniaturized specimens, is capable of withstanding a maximum axial force of 2.5 kN.

The preparation of the specimen is crucial in MTT because miniaturized specimens are more susceptible to large-dimensional deviations. Accordingly, in this work the specimen profile was obtained by wire electrical discharge machining (EDM) using a 0.25 mm wire diameter. This process is widely used for miniaturized specimen manufacturing, resulting in good dimensional accuracy and surface finishing [2].

### 2.3. Testing Conditions

In order to evaluate the monotonic behavior of DP500 and DP780 steels, tests were performed with tensile and compressive loading using miniaturized samples and the geometry shown in Figure 2b. Additionally, the same materials were tested on macro-tensile test pieces, in accordance with ISO 6892-1 [18]. These macro standard tensile tests were performed on a 300 kN Instron 5900 R testing machine. The experimental conditions for both geometries are shown in Table 2. The specimens were tested at a constant test speed, resulting in an initial strain rate of approximately $10^{-3}$ s${}^{-1}$, until the material failed. To ensure the repeatability of the results, two samples were systematically tested for each geometry.

### 2.4. Digital Image Correlation

#### 2.4.1. Choice of the Optical Technique

The DIC technique was coupled with the mechanical tests to quantify the deformation of the material across the central region of interest for both macro and miniaturized specimens, with a suitable spatial resolution and accuracy at the scale of observation. Traditionally, in carrying out experimental tests (e.g., macro tensile test [18]), the strain components are measured by means of a strain gauge attached to the sample. However, for miniaturized specimens, these instrumentation devices may not be suitable due to the reduced sample size. Nevertheless, a non-contact optical technique can be conveniently used to access full-field strains over the target region. For that purpose, several white-light and interferometric optical methods have been proposed in the literature [30]. Among them, DIC has been increasingly used in recent years in the field of experimental solid mechanics [31,32]. This technique was introduced in the 1980s [33,34] and allows the measurement of full-field kinematic quantities by tracking unique features on images taken at different deformation stages, typically by means of a subset-based approach [35,36,37,38]. The DIC technique uses a camera-lens optical setup, and it allows variable sensitivity and resolution in strain measurements. Since the DIC method uses digital images, several high spatial-resolution digital devices can be used for image acquisition. For example, DIC can be coupled with Optical Microscopy (OM) [39,40], Scanning Electron Microscopy (SEM) [41,42], and Scanning Tunneling Microscope (STM) [43] to realize microscale-to-nanoscale deformation measurements. In the 2D DIC set-up, the planar surface of a specimen is imaged stringently parallel to the camera sensor and the surface must have a random pattern of a contrasted speckle pattern. The creations of a quality pattern for DIC at reduced scales can be challenging [44], and the accuracy of the measurements depends significantly on the spatial resolution of the imaging system [35].

#### 2.4.2. Optical System and Speckle Pattern

A 2D DIC set-up using a 5 MP CMOS digital camera was selected in this work. The mounted lens was selected with regard to the specimen size. The details of the camera-lens system used for the miniaturized tests are summarized in Table 3. In this case, conversely, to a standard 50 mm focal lens (Fujinon HF50HB-1B 50 mm lens, f/2.3 C–Mount) used in macroscopic tensile tests, a telecentric lens was selected. It had the advantage of keeping the image magnification unchanged during the mechanical test, avoiding artifacts due to, for instance, Poisson effects or slightly out-of-plane movements during the test. In this case, the undeformed image was focused by fixing the working distance at 63.3 mm with a magnification factor of 1×. Images were recorded during the tests at an acquisition frequency of 5 Hz. The central part of the specimens was initially painted by spraying a thin layer of white paint in order to make the diffuse reflectivity of the surface uniform. The final speckle pattern was then created by spreading dark paint using an airbrush (Harder & Steenbeck—Evolution Silverline Solo) with a nozzle set of 0.2 mm. Considering the region of interest and camera-lens system, an average speckle size of about 6 pixels (21 µm) was obtained in the speckle quality evaluation. According to international guidelines [45], this means that a subset size larger than 21 pixels (0.075 mm) or 31 pixels (0.111 mm) must be used in the DIC measurements for accuracy.

#### 2.4.3. Selecting DIC Setting Parameters

In performing DIC measurements, a convergence analysis must be carried out for the inherent parameters in the numerical imaging method [29,46]. The set of DIC parameters plays an important role in the spatial resolution and accuracy associated with both displacement and strain measurements [47]. A parametric analysis was therefore carried out in this study, using the Performance Analysis Tool of the MatchID subset-based 2D DIC software [48]. The parametric tool allows one to carry out several DIC analyses over the same set of images by considering several combinations of parameters, as reported in Table 4. Each relevant parameter was considered over a suitable design space by defining the minimum and maximum values and a step of an integer increment (i.e., a subset size with a minimum of 21 × 21 pixels${}^{2}$, a maximum of 91 × 91 pixels${}^{2}$, and increment of 10 pixels, Table 4). Results in terms of signal evaluation at the center of the specimen can then be discussed in light of the preselected DIC setting parameters. The linear strain component in the *x* direction ($\varepsilon_{xx}$) was used as a reference, since uniaxial tests were carried out along this axis. Each set of DIC parameters can be associated with a corresponding spatial resolution. To quantify this metric, the following Virtual Strain Gauge (VSG) measure was used [45,48]:

(1) $$\mathrm{VSG}=\left[(\mathrm{SW}-1)\times \mathrm{ST}\right]+\mathrm{SS}\;\left[\mathrm{pixels}\right]$$

where SW stands for a strain window, i.e., the number of displacement data points used to fit a local polynomial in the neighbor of the subset centroid under evaluation; ST is the subset step; and SS is the subset size. The VSG can be expressed in physical units of millimeters if the image conversion factor of the optical system is taken into account (Table 3).

**Figure 3:**
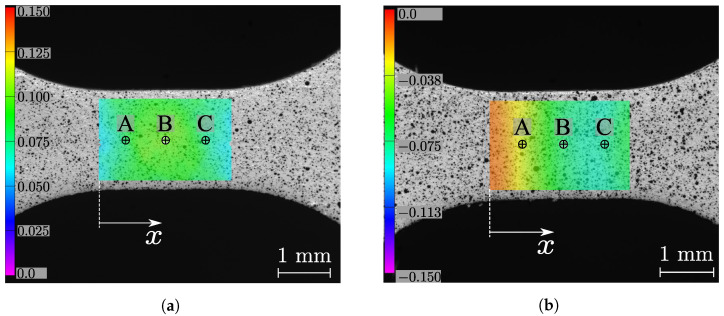
Points of reference used in evaluating the reconstruction of the $\varepsilon_{xx}$ strain signal with regard to the DIC settings and as a function of the VSG for both (**a**) tension and (**b**) compression tests.

In order to evaluate the $\varepsilon_{xx}$ strain signal with regard to the DIC settings as a function of the VSG, three points of reference at the centre of the specimens were systematically analyzed for both tensile and compression tests, for the DP500 steel, as schematically shown in Figure 3.

**Figure 4:**
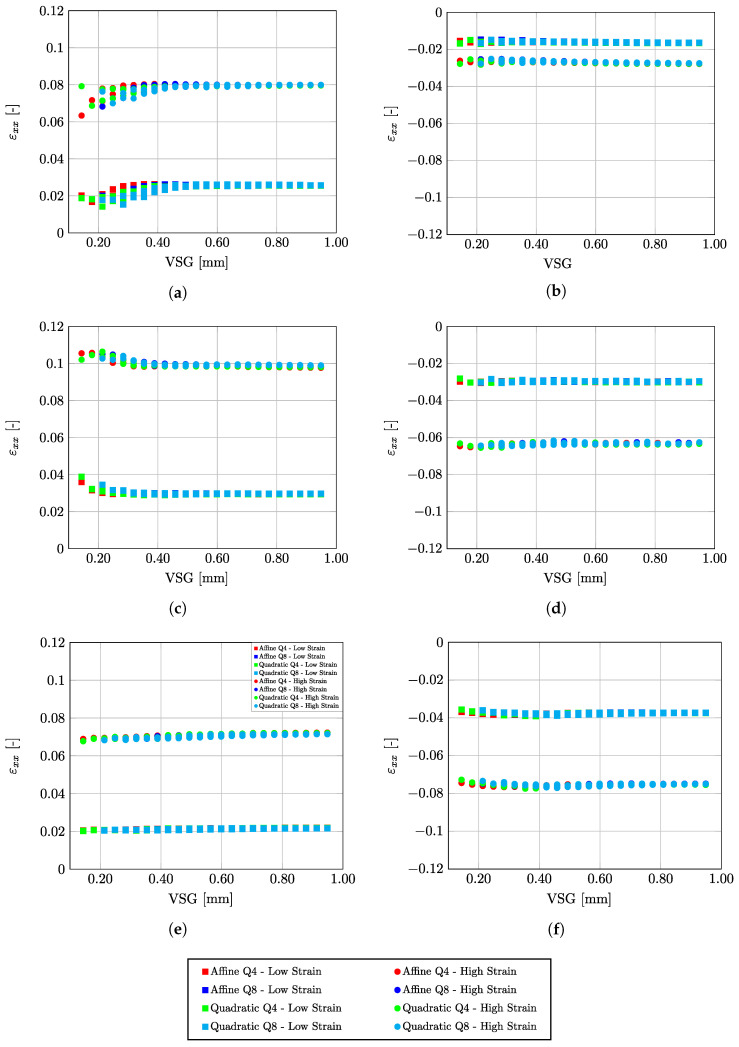
Evaluation of $\varepsilon_{xx}$ for points A, B, and C (Figure 3) as a function of DIC parameters expressed with regard to Virtual Strain Gauge (VSG) for both tension and compression tests, at two stages of deformation corresponding low and high strains. (**a**) Point A—Tension; (**b**) Point A—Compression; (**c**) Point B—Tension; (**d**) Point B—Compression; (**e**) Point C—Tension; (**f**) Point C—Compression.

**Figure 5:**
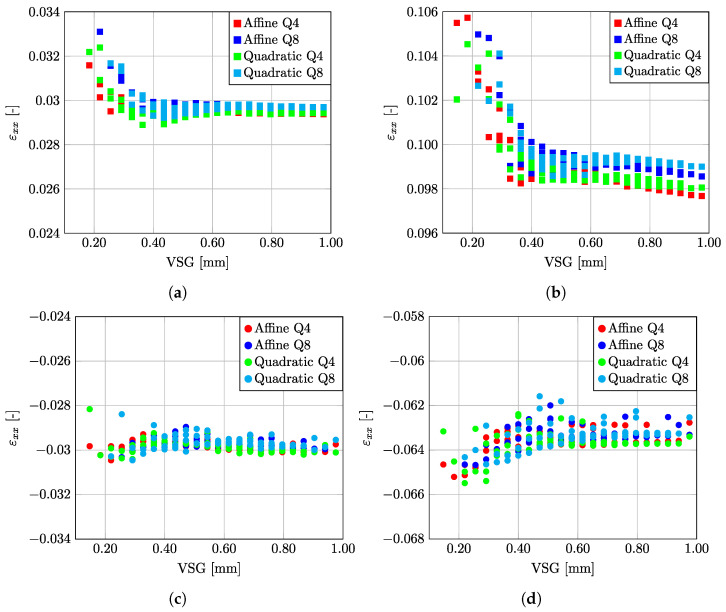
Evaluation of $\varepsilon_{xx}$ at point B (Figure 3) as a function of DIC parameters expressed with regard to Virtual Strain Gauge (VSG). (**a**) Tension-Low Strain; (**b**) Tension—High Strain; (**c**) Compression—Low Strain; (**d**) Compression—High Strain.

Figure 4 shows the evolution of $\varepsilon_{xx}$ as function of points A, B, and C (Figure 3) for both tensile and compression tests at two different deformation stages corresponding to low and high average strains, respectively. As can be observed, the DIC setting parameters converge systematically to an average reconstruction of the linear strain. In a more local analysis, Figure 5 shows the results of the reconstructed $\varepsilon_{xx}$ value at point B in the center of the specimen (see Figure 3) as a function of VSG, for the different DIC setting parameters for both tensile and compression tests and for two stages at low and high strain values, respectively. A good balance is to be found with regard to the spatial resolution in reconstructing the strain field. As can be seen in all scenarios, the signal is rather stable with regard to a set of parameters. This may be expected for a homogeneous material and uniaxial loading configuration.

**Figure 6:**
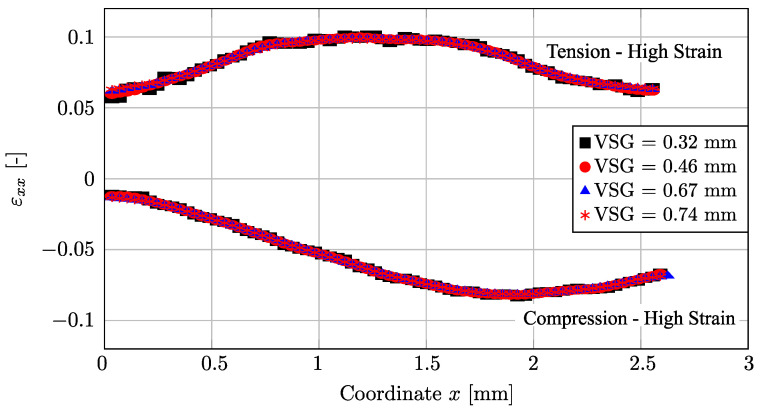
Evaluation of $\varepsilon_{xx}$ as a function of the coordinate *x* for several Virtual Strain Gauge (VVSG) values for both tension and compression tests.

Figure 6 shows a complementary analysis plotting the $\varepsilon_{xx}$ value along the coordinate *x* at the center of both the tensile and the compression tests for several VSG values. As can be seen, the DIC setting corresponding to higher values of VSG shows a smooth signal evaluation, as it may be expected, in contrast with lower VSG DIC settings. In conclusion, from the analysis in this work, the DIC settings reported in Table 5 were finally selected in a suitable balance between spatial resolution and accuracy of results for the current application.

## 3. Results and Discussion

### 3.1. Monotonic Tension and Compression Tests

**Figure 7:**
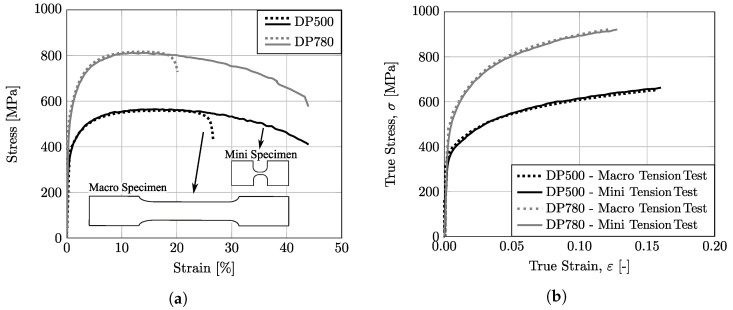
Experimental tensile stress/strain behavior for DP500 and DP780 steels: (**a**) engineering stress–strain curves; (**b**) true stress–strain curves.

For the reconstruction of the strain signal during the mechanical tests, an average value was systematically calculated at the centre of the specimen corresponding to a region of 2 mm × 2 mm. Figure 7 shows a representative stress–strain curve for both DP500 and DP780 steel materials. The engineering stress–strain curves, shown in Figure 7a, indicate that the results obtained from both macro and miniaturized specimens are quite comparable. In fact, for both materials, the work hardening is very similar for the mini and macro geometry, as seen in the true stress–true strain curve (σ−ε) shown in Figure 7b. However, it can be seen that after the point of ultimate tensile strength, the miniaturized geometry presents a superior total elongation when compared to the macro geometry. This difference can be justified by the local necking that occurs after reaching the point of maximum stress, which translates into a localized strain zone whose size is independent of the gauge length. Therefore, for smaller gauge lengths, the localized strain has a higher impact on the total elongation value. Additionally, the variation in necking behavior should be taken into account as well. As reported by [49], total elongation and post-necking elongation tend to increase with a decreasing ratio, w/t, between specimen width and thickness. In fact, when w/t decreases, the shear deformation may occur in both the upper (lower) and side planes, resulting in complex stress distribution, and the localized necking changes to diffuse necking, which leads to higher post-necking elongation.

**Figure 8:**
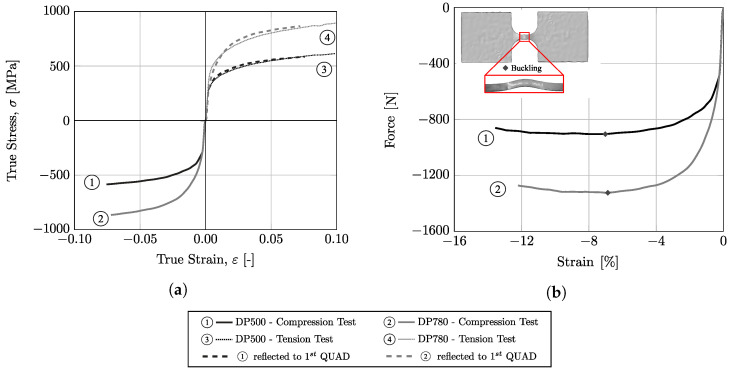
True stress–strain curves from monotonic compression tests and comparison with tension tests; (**b**) buckling point at the compression test.

The hardening behavior of the two dual-phase steels under compressive loading was also evaluated using the miniaturized specimens. The true stress–true strain curve (σ−ε) for each material is shown in Figure 8a. Additionally, this figure represents, for each material, the tensile hardening curve obtained using miniaturized samples (curve defined by points). For an easy comparison with these two tensile curves, the compression results were reflected for the first quadrant and represented as a dashed line. When analyzing results, it was verified that the work hardening was similar in the tension and compression for both materials; however, in terms of compression, the maximum strain values in the compression test were lower than those reached in tension. As seen in Figure 8b, for a strain value close to 7%, the force reaches a maximum value. At this point, the test becomes non-planar and the buckling phenomenon becomes evident. This instability is translated in the decay of the stress, which means that the test is not longer valid.

### 3.2. Reverse Loading Tests: Tension–Compression

**Figure 9:**
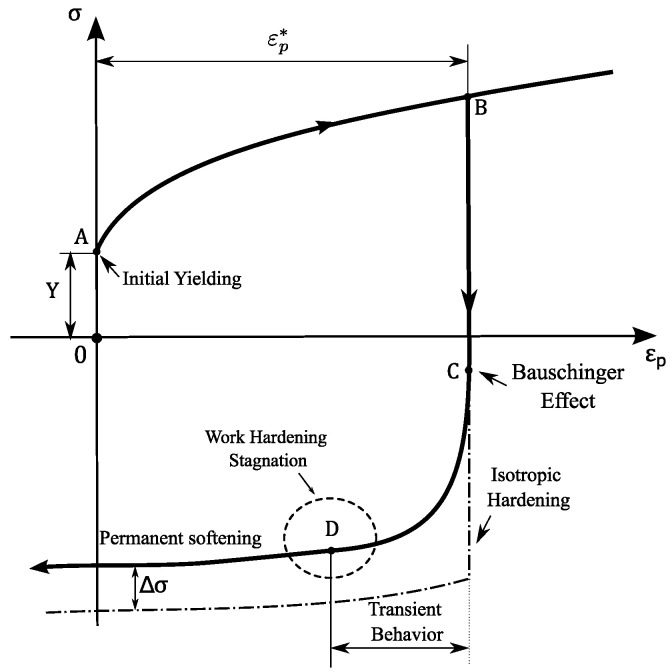
Schematic illustration of stress–strain response during forward and reverse deformation.

As represented in Figure 9, a typical hardening curve of a metallic material during reverse loading can be divided into four interconnected phenomena [50]: (a) the Bauschinger effect, which describes a reduction in the yield stress after the occurrence of plastic deformation during the initial loading; (b) the transient behavior, which is given by a smooth elasto-plastic transition characterized by a change in the work-hardening rate; (c) the work hardening stagnation, given by a transient behavior in which a stress–strain plateau is observed; (d) permanent softening, characterized by lower levels of stress after the transient period when compared to monotonic loading at a given accumulated plastic strain. These phenomena will hereafter be referred to as Bauschinger behavior. The experimental characterization of Bauschinger behavior is crucial to calibrate advanced constitutive models, e.g., the multi-surface Yoshida–Uemori (Y–U) [51] kinematic hardening model and the Homogeneous anisotropic hardening (HAH) model proposed by [52], and consequently improves the accuracy of numerical simulation results of sheet-metal-forming processes. Different authors have shown that the Bauschinger behaviour has a strong influence on the prediction of the springback [53,54,55]. This phenomenon, which refers to the elastic distortion of a part when forming tools are removed, is a major issue in sheet metal forming processes and its mitigation is crucial.

**Figure 10:**
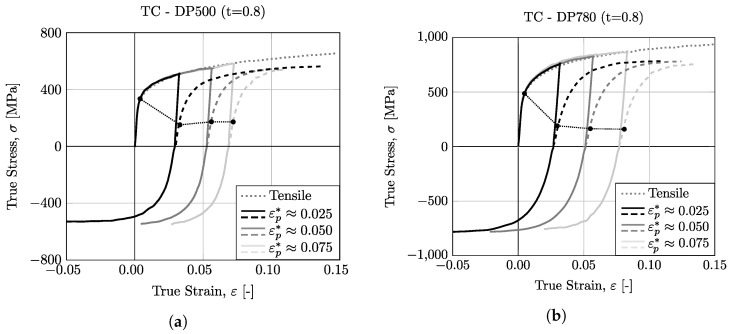
True Stress–Strain curves from tension–compression tests for (**a**) DP500 and (**b**) DP780.

**Figure 11:**
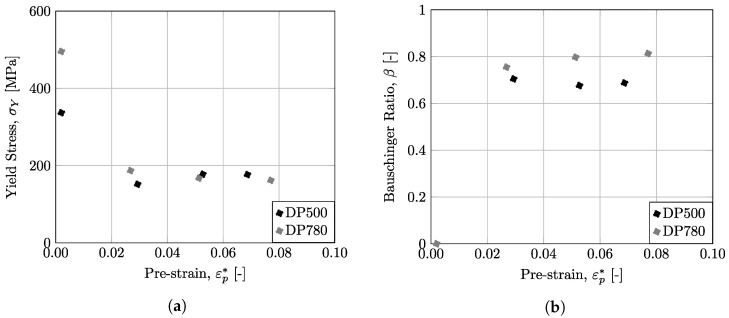
(**a**) Yield stress, $\sigma_{Y}$, and (**b**) Bauschinger Ratio, β, values for different pre-strains, $\varepsilon_{p}^{*}$.

In this work, the Bauschinger behavior of the DP500 and DP780 materials was evaluated through uniaxial tests with reverse loading (tension–compression) using miniaturized specimens. The test includes a step of tensile loading, followed by a compression step. Initially, the tensile step is performed up to a defined pre-strain, $\varepsilon_{p}^{*}$. In this sense, three pre-strain values were selected, 0.025, 0.050, and 0.075. After this pre-strain in tensile loading, the compressive step was carried out until the buckling phenomenon began to be evident, which is shown, as already mentioned, by a decay of the stress value in the stress–strain curve. The results for DP500 and DP780 steels are shown in Figure 10a,b, respectively. For each material, the monotonic uniaxial tensile curve is also represented for comparison purposes. It can be seen that the tension step of tension–compression test matches the uniaxial tensile curve very well, for both materials, which confirms that the equipment provide excellent repeatability of results. The compression step curves for the three different pre-strain values show a similar changing trend across the entire strain range, indicating that the device can provide reliable results for the studied materials. To analyze the Bauschinger behavior of the dual-phase steels used, the compression-step-hardening curves were reflected to the first quadrant, as shown in the dashed line in Figure 10. The yield point for the uniaxial tensile curve and the reflected reverse loading curves, represented in round points, were taken at the 0.2% plastic strain and are summarized in Figure 11a. It can be observed that the studied materials present a strong Bauschinger effect, since the reverse yield stress is significantly reduced during reverse loading. A strong permanent softening effect is also visible, since the curves do not coincide with the monotonic tensile curve and tend to be approximately parallel.

To quantify the Bauschinger effect, several indicators have been proposed over the years. One of the most used, the so-called Bauschinger ratio, β, is based on stress values and is expressed mathematically by Equation (2):

(2) $$\beta =\frac{\sigma_{max}^{T}+\sigma_{Yield}^{C}}{\sigma_{max}^{T}}$$

where $\sigma_{max}^{T}$ represents the maximum stress in the forward load (tensile step) and $\sigma_{Yield}^{C}$ represents the yield stress in the backwards or reverse load (compression step)-point B and C, respectively, in Figure 9. The Bauschinger ratio quantifies the magnitude of the Bauschinger effect. The higher the parameter, the greater the Bauschinger effect of the material. In the case of isotropic hardening, the Bauschinger ratio is equal to zero. The Bauschinger ratio for the materials under this study is represented in Figure 11b, which is dependent on the applied pre-strain. From these results, it is possible to observe that both materials have a strong Bauschinger effect, since whenever the loading direction is reversed, the yield stress is reduced by about 70–80% compared to the value of the stress in the opposite direction.

## 4. Conclusions

This work presents the development of equipment dedicated to performing uniaxial, tensile–compression tests on miniaturized metallic specimens. The experimental equipment, called MSTD (Miniaturized Specimen Tester Device), allows the testing of specimens on a miniaturized scale, which opens the field of applications, one of them being compressive loading tests, having the advantage of overcoming buckling effects. In addition to uniaxial tensile, compression, and cyclic tests, it is also possible and expected that equipment will adapt to other fundamental tests for metallic sheets, such as shear and ductile damage tests.

The digital image correlation (DIC) technique was used to capture the full-field strain in the gauge section of the sample. A sensitivity analysis of the DIC setting parameters permitted to quantify the influence on results for subset size, subset step, strain window, shape function, and polynomial order. These three first parameters are included in the Virtual Strain Gage variable (VSG) and this variable was tested for different scenarios in tensile and compressive loading, giving a rather stable signal and a fast convergence of results. A good balance was found using a VSG of 151 px, which corresponds to 0.53 mm, thus giving the best ratio for efficiency and accuracy of results.

In order to validate the developed solution, several experimental tests were carried out using two dual-phase steels: DP500 and DP780. The tests performed on the developed equipment allowed the comparison of results for different types of loadings. Initially, monotonic tensile tests were carried out, and the results obtained with the miniaturized specimens were shown to be quite similar to those obtained by using the standardized geometry of tensile tests at the macro scale. The results of the compression tests showed that it is possible, with the developed equipment, to characterize materials up to 7.5% strain in compressive loading, without buckling, thus creating the possibility of sheet metal characterization for stress differential and Bauschinger behavior. Additionally, it was seen that the work-hardening of tested dual-phase steels (DP500, DP780) in compressive loading is very similar to their work hardening in tensile loading. Finally, uniaxial tests with reverse loadings showed that both DP500 and DP780 present a strong Bauschinger effect, since the yield stress undergoes a considerable decrease with reverse loading. 

## Figures and Tables

**Table 1:** Chemical composition of the DP500 and DP780 dual-phase steels.

Element [%]	C	Si	Mn	P	S	Cr	Ni	V	Cu	Al	Nb	B	N	EC ^1^
DP500	0.079	0.31	0.65	0.003	0.003	0.03	0.03	0.01	0.01	0.038	0.0	0.0003	0.003	0.20
DP780	0.138	0.20	1.52	0.011	0.002	0.03	0.03	0.02	0.01	0.038	0.014	0.0002	0.003	0.40

^1^ EC = *C* + $\frac{\mathrm{Mn}}{6}+\frac{\mathrm{Ni}+\mathrm{Cu}}{15}+\frac{\mathrm{Cr}+\mathrm{Mo}+V}{5}$

**Table 2:** Experimental conditions for macro and miniaturized samples.

	Macro Sample	Miniaturized Sample
Number of samples	2	2
Gauge length ($L_{0}$)	50 mm	2 mm
Crosshead speed	5 mm/min	0.5 mm/min
Frequency of data acquisition	20 Hz	20 Hz
Temperature	23 °C	23 °C
Humidity	53%	53%

**Table 3:** Camera-lens optical system for the miniaturized tests.

Camera	Basler acA2440-75um, 5 MPixel, CMOS sensor
Pixel resolution	2448 px × 2048 px
Lens	Opto Engineering TC 23 09
Field of view	8.8 mm × 6.6 mm
Magnification factor	1×
Working distance	63.3 mm
Image-conversion factor	3.5 μm/px
Image-acquisition frequency	5 Hz
Speckle pattern technique	Airbrush (nozzle set of 0.2 mm)
Average speckle size	6 px|21.5 μm

**Table 4:** Parameters used in the Performance Analysis Tool of MatchID 2D DIC.

Subset-Based Settings	
Subset size	SS ∈{21,31,41,51,61,71,81,91}px
Subset step	ST = 10 px (fixed)
Shape function	{Affine, Quadratic}
**Strain reconstruction-based settings**	
Strain window	SW ∈{3,5,7,11,13,15,17,19}datapoints
Polynomial order *	Bilinear (Q4), Biquadratic (Q8)
Strain convention	Green–Lagrange

* Local least-squares fitting approach for the strain evaluation.

**Table 5:** DIC and strain settings selected in the full-field measurements.

DIC Settings	
Correlation criterion	ZNSSD
Interpolant	Bicubic spline
Subset shape function	Affine
Subset size	71 px
Step size	10 px
Image pre-filtering	Gaussian, 5 px kernel
**Strain Settings**	
Strain window size	9 data points
Strain interpolation	Bilinear Q4
Strain convention	Green–Lagrange

## Data Availability

Not applicable.

## Abbreviations

The following abbreviations are used in this manuscript:

CCD	Charge-Coupled Device
DIC	Digital Image Correlation
EDM	Electrical Discharge Machining
ESPI	Electronic Speckle-Pattern Interferometry
FEM	Finite Element Method
HAH	Homogeneous Anisotropic Hardening
MEMS	Microelectromechanical systems
MSTD	Miniaturized Specimen Tester Device
MTT	Miniaturized Tensile Test
OM	Optical Microscopy
SEM	Scanning Electron Microscopy
SS	Subset Size
SSTT	Small Specimen Test Technology
ST	Subset Step
STM	Scanning Tunneling Microscope
SW	Strain Window
VSG	Virtual Strain Gauge

## References

[1] Gorji M.B., Furmanski J., Mohr D. (2021). From macro- to micro-experiments: Specimen-size independent identification of plasticity and fracture properties. Int. J. Mech. Sci..

[2] Zheng P., Chen R., Liu H., Chen J., Zhang Z., Liu X., Shen Y. (2020). On the standards and practices for miniaturized tensile test—A review. Fusion Eng. Des..

[3] Lucon E., Hashmi S., Batalha G.F., Van Tyne C.J., Yilbas B. (2014). 1.08-Testing of Small-Sized Specimens. Comprehensive Materials Processing.

[4] Klueh R. (1985). Miniature tensile test specimens for fusion reactor irradiation studies. Nucl. Eng. Des. Fusion.

[5] Şahin S., Saeed A. (2016). Experimental evaluation of surveillance capsule assemblies for life assessment of CHASNUPP Unit-1 reactor pressure vessel. Ann. Nucl. Energy.

[6] Lucon E. (2001). Material damage evaluation and residual life assessment of primary power plant components using specimens of non–standard dimensions. Mater. Sci. Technol..

[7] Oluwasegun K., Cooper C., Chiu Y., Jones I., Li H., Baxter G. (2014). Micro-tensile strength of a welded turbine disc superalloy. Mater. Sci. Eng. A.

[8] Liu P., Bao J., Bao Y. (2019). Mechanical Properties and Fracture Behavior of an EBW T2 Copper–45 Steel Joint. Materials.

[9] Gil J., Seca R., Amaral R., Emadinia O., Reis A., Jesus A. (2022). 18Ni300 Maraging Steel Produced via Direct energy Deposition on H13 Tool Steel and DIN CK45. Key Engineering Materials.

[10] Azinpour E., Cruz D.J., de Sa J.M.A.C., Santos A. (2021). Phase-field approach in elastoplastic solids: Application of an iterative staggered scheme and its experimental validation. Comput. Mech..

[11] Spearing S. (2000). Materials issues in microelectromechanical systems (MEMS). Acta Mater..

[12] Rajagopalan J., Schmauder S., Chen C.S., Chawla K.K., Chawla N., Chen W., Kagawa Y. (2019). Microelectromechanical Systems (MEMS)-Based Testing of Materials. Handbook of Mechanics of Materials.

[13] Rund M., Procházka R., Konopík P., Džugan J., Folgar H. (2015). Investigation of Sample-size Influence on Tensile Test Results at Different Strain Rates. Procedia Eng..

[14] Read D.T., Liew L.A., White R.M., Barbosa N., Geaney J. (2019). Measuring flow curve and failure conditions for a MEMS-scale electrodeposited nickel alloy. Mater. Res. Express.

[15] Hyun H.C., Kim M., Bang S., Lee H. (2014). On acquiring true stress–strain curves for sheet specimens using tensile test and FE analysis based on a local necking criterion. J. Mater. Res..

[16] Kamaya M., Kitsunai Y., Koshiishi M. (2015). True stress–strain curve acquisition for irradiated stainless steel including the range exceeding necking strain. J. Nucl. Mater..

[17] Hattalli V.L., Srivatsa S.R. (2018). Sheet Metal Forming Processes–Recent Technological Advances. Mater. Today Proc..

[18] (2019). Metallic Materials—Tensile Testing—Part 1: Method of Test at Room Temperature.

[19] (2022). Metallic Materials—Sheet and Strip—Determination of Biaxial Stress-Strain Curve by Means of Bulge Test with Optical Measuring Systems.

[20] Hetz P., Kraus M., Merklein M. (2022). Characterization of sheet metal components by using an upsetting test with miniaturized cylindrical specimen. CIRP Ann..

[21] Aitchison C., Tuckerman L. (1939). The Pack Method for Compressive Tests of Thin Specimens of Materials Used in Thin-Wall Structures.

[22] Jackman K. (1944). Improved methods for determining the compression properties of sheet metal. Automot. Aviat. Ind..

[23] Yoshida F., Uemori T., Fujiwara K. (2002). Elastic–plastic behavior of steel sheets under in-plane cyclic tension–compression at large strain. Int. J. Plast..

[24] Boger R.K., Wagoner R.H., Barlat F., Lee M.G., Chung K. (2005). Continuous, large strain, tension/compression testing of sheet material. Int. J. Plast..

[25] Sekine A., Kuwabara T. (2005). 616 Development of In-Plane Reverse Loading Test Apparatus and Measurement of the Bauschinger Effect of Sheet Metals. Proc. Autumn Conf. Tohoku Branch.

[26] Tritschler M., Butz A., Helm D., Falkinger G., Kiese J. (2014). Experimental analysis and modeling of the anisotropic response of titanium alloy Ti-X for quasi-static loading at room temperature. Int. J. Mater. Form..

[27] Hußnätter W. Yielding of magnesium alloy AZ31. Proceedings of the ICTP2008.

[28] Cruz D.J., Shamchi S.P., Santos A.D., Amaral R.L., Tavares P.J., Moreira P. (2020). Development of a mini-tensile approach for sheet metal testing using Digital Image Correlation. Proc. Struct. Integr..

[29] Pereira J., Xavier J., Ghiassi B., Lousada J., Morais J. (2018). On the identification of earlywood and latewood radial elastic modulus of Pinus pinaster by digital image correlation: A parametric analysis. J. Strain Anal. Eng. Des..

[30] Grédiac M., Hild F. (2012). Full-Field Measurements and Identification in Solid Mechanics.

[31] Pan B. (2018). Digital image correlation for surface deformation measurement: Historical developments, recent advances and future goals. Meas. Sci. Technol..

[32] Cunha F., Santos T., Xavier J. (2021). In Situ Monitoring of Additive Manufacturing Using Digital Image Correlation: A Review. Materials.

[33] Sutton M., Mingqi C., Peters W., Chao Y., McNeill S. (1986). Application of an optimized digital correlation method to planar deformation analysis. Image Vis. Comput..

[34] Peters W.H., Ranson W.F. (1982). Digital Imaging Techniques in Experimental Stress Analysis. Opt. Eng..

[35] Pan B., Qian K., Xie H., Asundi A. (2009). Two-dimensional digital image correlation for in-plane displacement and strain measurement: A review. Meas. Sci. Technol..

[36] Xavier J., de Jesus A., Morais J., Pinto J. (2012). Stereovision measurements on evaluating the modulus of elasticity of wood by compression tests parallel to the grain. Constr. Build. Mater..

[37] Sousa A.M.R., Xavier J., Vaz M., Morais J.J.L., Filipe V.M.J. (2011). Cross-Correlation and Differential Technique Combination to Determine Displacement Fields. Strain.

[38] Sendrowicz A., Myhre A.O., Wierdak S.W., Vinogradov A. (2021). Challenges and Accomplishments in Mechanical Testing Instrumented by In Situ Techniques: Infrared Thermography, Digital Image Correlation, and Acoustic Emission. Appl. Sci..

[39] Zhang D., Luo M., Arola D. (2006). Displacement/strain measurements using an optical microscope and digital image correlation. OPT Eng..

[40] Sun Z., Lyons J.S., McNeill S.R. (1997). Measuring Microscopic Deformations with Digital Image Correlation. Opt. Lasers Eng..

[41] Sabaté N., Vogel D., Gollhardt A., Keller J., Michel B., Cané C., Gràcia I., Morante J.R. (2006). Measurement of residual stresses in micromachined structures in a microregion. Appl. Phys. Lett..

[42] Touchard F., Bridier F., Villechaise P., Brillaud J. (2006). In-plane strain measurements on a microscopic scale by coupling digital image correlation and an in situ SEM technique. Mater. Charact..

[43] Vendroux G., Knauss W. (1998). Submicron deformation field measurements: Part 1. Developing a digital scanning tunneling microscope. Exp. Mech..

[44] Dong Y., Pan B. (2017). A Review of Speckle Pattern Fabrication and Assessment for Digital Image Correlation. Exp. Mech..

[45] Jones E., Iadicola M. (2018). A Good Practices Guide for Digital Image Correlation.

[46] Henriques J., Xavier J., Andrade-Campos A. (2022). Identification of Orthotropic Elastic Properties of Wood by a Synthetic Image Approach Based on Digital Image Correlation. Materials.

[47] Lava P., Jones E., Wittevrongel L., Pierron F. (2020). Validation of finite-element models using full-field experimental data: Levelling finite-element analysis data through a digital image correlation engine. Strain.

[48] MatchID (2017). MatchID Manual.

[49] Yuan W., Zhang Z., Su Y., Qiao L., Chu W. (2012). Influence of specimen thickness with rectangular cross-section on the tensile properties of structural steels. Mater. Sci. Eng. A.

[50] Bauschinger J. (1886). On the change of the position of the elastic limit of iron and steel under cyclic variations of stress. Mitt. Mech. Tech. Lab. Munich.

[51] Yoshida F., Uemori T. (2002). A model of large-strain cyclic plasticity describing the Bauschinger effect and workhardening stagnation. Int. J. Plast..

[52] Barlat F., Gracio J.J., Lee M.G., Rauch E.F., Vincze G. (2011). An alternative to kinematic hardening in classical plasticity. Int. J. Plast..

[53] Lee M.G., Kim D., Kim C., Wenner M.L., Chung K. (2005). Spring-back evaluation of automotive sheets based on isotropic–kinematic hardening laws and non-quadratic anisotropic yield functions, part III: Applications. Int. J. Plast..

[54] Oliveira M., Alves J., Chaparro B., Menezes L. (2007). Study on the influence of work-hardening modeling in springback prediction. Int. J. Plast..

[55] Yoshida F. Material models for accurate simulation of sheet metal forming and springback. Proceedings of the AIP Conference Proceedings, American Institute of Physics.

